# Primary Retroperitoneal Mucinous Cystic Neoplasm Involving Adrenal Gland

**DOI:** 10.1002/kjm2.70181

**Published:** 2026-02-04

**Authors:** Di‐Ping Yu, Xiao‐Long Liu, Xin‐Yu Li

**Affiliations:** ^1^ Department of Pathology The Puer People's Hospital Puer Yunnan Province China; ^2^ Department of Radiology The Puer People's Hospital Puer Yunnan Province China

Mucinous cystic neoplasm (MCN) is a cystic neoplasm with mucinous epithelium surrounded by ovarian‐like stroma. Extraovarian MCN occurring in the liver and pancreas has been well characterized. Primary retroperitoneal MCN is uncommon. Here, we report a case of retroperitoneal MCN with low‐grade intraepithelial dysplasia and involving the adrenal gland.

The patient was a 26‐year‐old female with no history of pregnancy or marriage, but having sex. She denied any family history of genetic disorders or cancer. Two weeks ago, she presented with bloating, mid‐epigastric pain, and nausea without vomiting, and mistakenly thought she was pregnant. The serum human chorionic gonadotropin (HCG) was normal in the laboratory. An abdominal computed tomography (CT) confirmed a cystic mass with a size of 6.1 cm, which was located in the left adrenal region, and the left kidney and spleen were compressed and displaced to varying degrees (Figure 1A). The abdominal ultrasound (US) showed a 5.8‐cm well‐defined solitary cystic mass in the left adrenal region, which contained multiple cystic cavities, smooth cystic walls, and no adnexal nodules, and the fluid in the cystic cavity was hypoechoic (Figure 1B). There were no abnormal findings in the bilateral ovaries, liver, and pancreas. The levels of tumor markers were within normal limits. The neoplastic nature of the mass was uncertain. Therefore, the patient underwent complete laparoscopic resection of a 6‐cm cyst without its disruption, and part of the adhered adrenal tissue was removed. A gross examination of the surgically resected specimen showed a multilocular cyst (5.9 cm × 4.4 cm × 3.5 cm) containing mucinous material, and the inner surface of the cyst was smooth with no mural nodules or papillae (Figure 1C). Microscopic examination showed the cyst was lined by tall columnar tumor cells possessing supranuclear cytoplasmic mucin with elongated nuclei and mild cytologic atypia. Ovarian‐like stroma cells could be seen under the epidermis (Figure 1D). On immunohistochemistry, the epithelium was positive for cytokeratin 7 (Figure 1E) and cytokeratin 19, while the stroma expressed estrogen receptor (ER) (Figure 1F) and progesterone receptor (PR). Based on these histologic and immunohistochemical findings, the diagnosis of MCN was rendered. The patient was followed up for 15 months after the operation, and there was no sign of disease recurrence.

**FIGURE 1:**
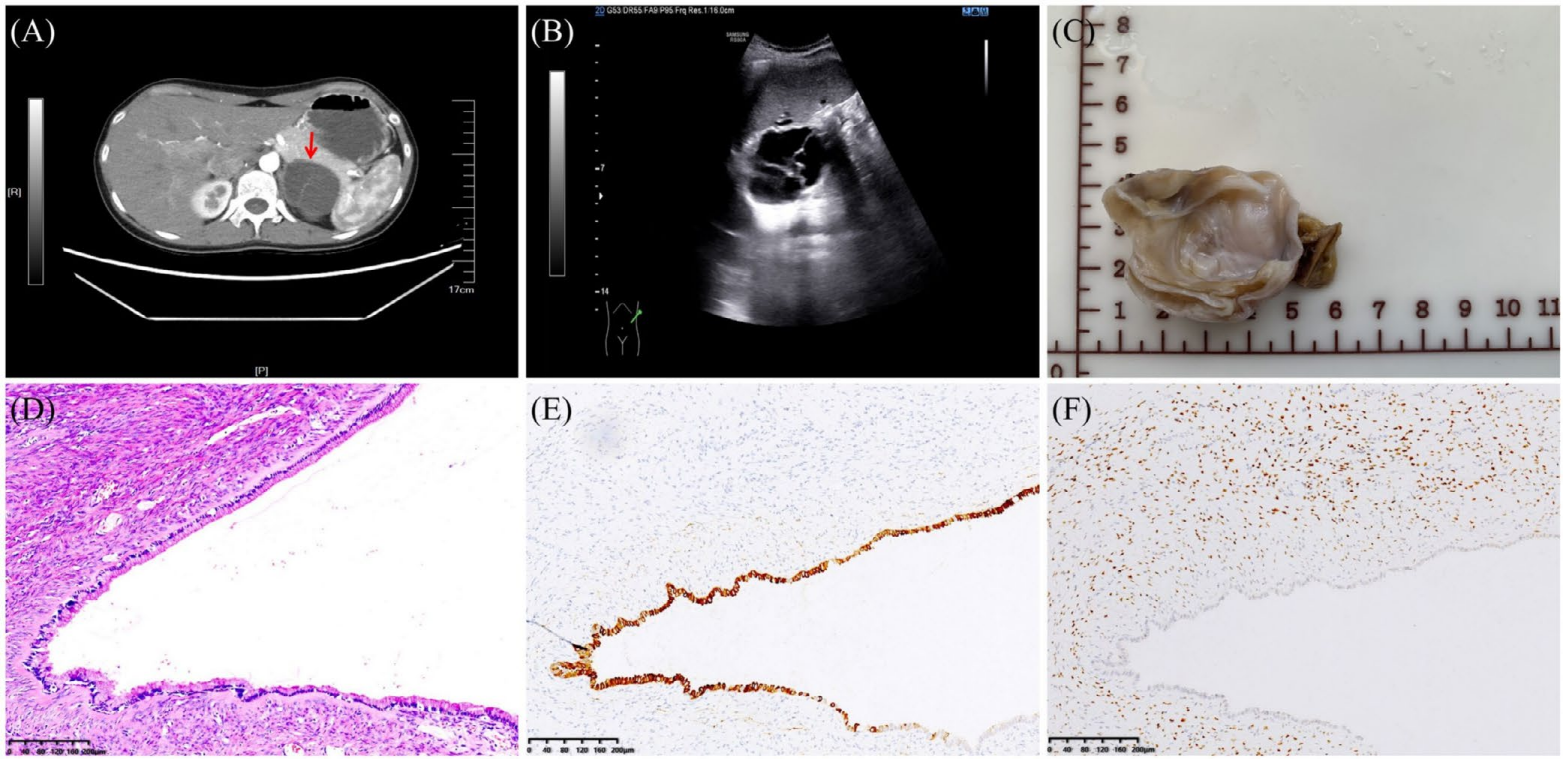
(A) An abdominal computed tomography (CT) confirmed a cystic mass with a size of 6.1 cm × 5.6 cm × 4.6 cm, which was located in the left adrenal region (red arrow). (B) The abdominal ultrasound (US) showed a 5.8‐cm well‐defined solitary cystic mass in the left adrenal region, which contained multiple cystic cavities, smooth cystic walls, and no adnexal nodules. (C) A gross examination of the surgically resected specimen showed a multilocular cyst containing mucinous material, and the inner surface of the cyst was smooth with no mural nodules or papillae. (D) Microscopic examination showed the cyst was lined by tall columnar tumor cells possessing supranuclear cytoplasmic mucin with elongated nuclei and mild cytologic atypia. Ovarian‐like stroma cells could be seen under the epidermis (H&E, original magnification ×100). (E) The epithelium was positive for cytokeratin 7. (F) The stroma cells were positive for ER.

Primary retroperitoneal MCN is a rare, unilocular or multilocular cystic tumor, with histologic or clinical confirmation of the lack of ovarian involvement. Overlapping features suggest a common histogenesis for all MCN, which could include periductal fetal mesenchyme, aberrant migration of primordial germ cells, or abnormal differentiation or metaplasia of the embryonic coelomic epithelium [1, 2]. This tumor occurs almost exclusively in women, with a mean age at presentation in the fourth decade. The occurrence of MCN in men is exceedingly rare [3]. Among the resected retroperitoneal tumors, the prevalence rate of primary retroperitoneal MCN was 1.95%. Symptoms vary according to the location of the tumor, but they are usually acute or chronic epigastric pain, nausea, and vomiting. For tumors with a diameter less than 4 cm, there are generally no obvious symptoms [4]. The laboratory tests are usually nonspecific, with no known specific tumor markers or radiological findings. Therefore, the preoperative diagnosis of primary retroperitoneal MCN is challenging, and the diagnosis depends on pathological examination. Histologically, MCN consists of cuboidal to columnar, variably mucin‐producing epithelium, associated with ovarian‐type subepithelial stroma and positive staining for estrogen and progesterone receptors [2]. According to the atypia of epithelial cells, MCN is graded as low‐grade, high‐grade intraepithelial dysplasia, and associated invasive carcinoma. In this case, the epithelial cells were only slightly atypical, and the pathological diagnosis was MCN with low‐grade intraepithelial dysplasia. The differential diagnosis should include metastatic mucinous tumors from the gastrointestinal tract, liver, and pancreas, which are more common than retroperitoneal MCN. The presence of ovarian‐type stroma in the histopathological section was established by the WHO classification as a diagnostic criterion for MCN.

Primary retroperitoneal MCN has a benign biological behavior. A 10‐year disease‐free survival (DFS) rate of 99% has been reported in patients with MCN [5]. The treatment of choice is complete tumor resection without cyst rupture to prevent tumor cell dissemination, and surgical resection of retroperitoneal MCNs appears to be curative, with no deaths reported in the literature to date.

## Conflicts of Interest

The authors declare no conflicts of interest.

## Data Availability

Data sharing is not applicable to this article as no datasets were generated or analyzed during the current study.
